# Supplementary material for: Causal Relationship between Aging and Anorexia Nervosa: A White-Matter-Microstructure-Mediated Mendelian Randomization Analysis
Source: Biomedicines. 2024 Aug 16;12(8):1874. doi: 10.3390/biomedicines12081874 (PMC11351342; doi:10.3390/biomedicines12081874)
Supplement: Supplementary file 1 [file biomedicines-12-01874-s001.zip › Supplementary Figure/Figure S8/Leaveoneout plot of MR analysis for TL to ubm-b-1517.pdf]

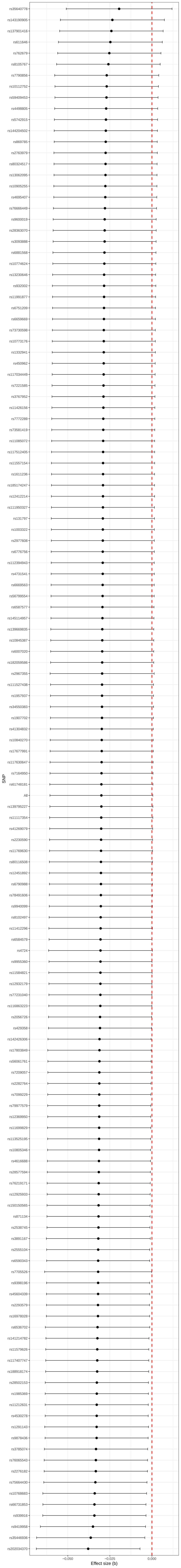

SNP
rs35640778
rs143190905
rs137901416
rs611646
rs762679
rs8105767
rs7790856
rs10112752
rs59409453
rs4498805
rs5742915
rs144204502
rs869785
rs2763979
rs80324517
rs13062095
rs10905255
rs4695407
rs76666449
rs9600019
rs28363070
rs3093888
rs6881568
rs10774624
rs13230646
rs932002
rs11991877
rs6751209
rs6659669
rs73730598
rs10773176
rs1332941
rs450962
rs117034449
rs7221585
rs3767952
rs11426156
rs7772289
rs73581419
rs11085072
rs117512405
rs11557154
rs1611236
rs185174247
rs12412214
rs111950327
rs131797
rs1003322
rs2977608
rs6776756
rs112394943
rs4731541
rs6669563
rs56799554
rs6587577
rs145114957
rs139669835
rs10845387
rs6007020
rs182059586
rs2967355
rs111527438
rs1957937
rs34550383
rs1907702
rs41304832
rs10840270
rs17677991
rs117630647
rs7164950
rs61748181
All
rs139795227
rs11117354
rs41269079
rs2230590
rs11769630
rs80116508
rs12451892
rs6790988
rs78491606
rs9940099
rs8102497
rs11412296
rs6584579
rs4724
rs9955360
rs11584821
rs12932179
rs77231040
rs116863223
rs2056726
rs429358
rs142426306
rs17803849
rs56061761
rs7209057
rs2282764
rs7099229
rs79977579
rs12369950
rs11699829
rs113525195
rs10805346
rs4616688
rs28577594
rs76219171
rs12925933
rs150150565
rs871134
rs2538745
rs3891167
rs2555104
rs6590343
rs7705526
rs9398196
rs45604339
rs2293579
rs16978028
rs6536702
rs141214782
rs11579626
rs117407747
rs188918174
rs28502153
rs1985369
rs11212631
rs4530278
rs1291143
rs9878436
rs3785074
rs76065543
rs2276182
rs75664430
rs10768683
rs66731853
rs939916
rs9419958
rs35446936
rs202034370
−0.050
−0.025
0.000
Effect size (b)
